# Maladie vectorielle à tiques chez un randonneur en Guyane : un cas d’anaplasmose présumée

**DOI:** 10.48327/mtsi.v6i1.2026.826

**Published:** 2026-03-13

**Authors:** Morgane BOURNE-WATRIN, Kinan DRAK ALSIBAI, Olivier DURON, Justin DESTOOP, Maylis DOUINE, Loïc EPELBOIN, Pierre COUPPIÉ

**Affiliations:** 1 Service de dermatologie-vénérologie, Centre hospitalier de Cayenne, Cayenne, France; 2 Laboratoire d’anatomie et cytologie pathologique, Centre hospitalier de Cayenne, Cayenne, France; 3 MIVEGEC (Maladies infectieuses et vecteurs : écologie, génétique, évolution et contrôle), Centre national de la recherche scientifique (CNRS) - Institut pour la recherche et le développement (IRD) - Université de Montpellier (UM), Montpellier, France; 4 Centre d’investigation clinique Antilles-Guyane CIC Inserm 1424, Centre hospitalier de Cayenne, Cayenne, France; 5 Unité des maladies infectieuses et tropicales, Centre hospitalier de Cayenne, Cayenne, France; 6 TBIP, Université de Guyane, Université de Lille, CNRS, Inserm, Institut Pasteur de Lille, U1019-UMR9017-CIIL Centre d’infection et d’immunité de Lille, Guyane française, France

**Keywords:** Anaplasmose, Maladie vectorielle à tique, Zoonose, Forêt amazonienne, Guyane française, Anaplasmosis, Tick-borne disease, Zoonoses, Amazon rainforest, French Guiana

## Abstract

**Introduction:**

De nouvelles espèces de *Borrelia, Rickettsia* et *Anaplasma* ont été récemment détectées chez des tiques en Guyane, et une nouvelle espèce d’*Anaplasma* a été découverte chez un homme. Des génovariants spécifiques d’*Anaplasma* et d’*Ehrlichia* ont également été détectés avec un cycle sylvatique propre en forêt amazonienne guyanaise.

**Présentation du cas:**

Nous rapportons le cas d’un patient ayant présenté de la fièvre, des myalgies, une éruption cutanée et des escarres d’inoculation après avoir subi des dizaines de morsures de tiques lors d’une randonnée en forêt amazonienne profonde. Les résultats biologiques retrouvaient une lymphopénie, une polynucléose neutrophile, une cytolyse et une cholestase hépatiques, ainsi qu’une protéine C-réactive à 78 mg/l. Les sérologies VIH, hépatites A, B, C et E, cytomégalovirus, syphilis, fièvre Q étaient négatives. L’antigène NS1, les PCR dengue et leptospirose étaient également négatifs. La biopsie cutanée d’une papule ulcéro-croûteuse de la jambe retrouvait un infiltrat inflammatoire dermique polymorphe avec un bacille Gram-négatif intramacrophagique. Les PCR sanguines pour *Anaplasma phagocytophilum, Borrelia, Rickettsia* et *Babesia* étaient négatives. Les sérologies étaient négatives pour *Rickettsia conorii* et *R. typhi*, mais positives pour *A. phagocytophilum* avec des IgM faiblement positives et des IgG positives, confirmées en sérologie de contrôle. Le patient s’est amélioré grâce à un traitement par doxycycline.

**Discussion et conclusion:**

Les nouvelles espèces d’*Anaplasma* et d’*Ehrlichia* détectées en forêt amazonienne guyanaise présentent des caractéristiques différentes des autres espèces de l’hémisphère Nord et sont difficiles à diagnostiquer avec des outils moléculaires non conçus pour détecter des agents pathogènes différents des espèces connues. Même si non prouvée par biologie moléculaire, cette observation peut être une anaplasmose ou une autre infection du groupe des Rickettsiales. Elle rappelle que l’on sait peu du potentiel des maladies vectorielles à tiques en Guyane. Dans ce contexte, les recommandations de protection antivectorielle doivent s’étendre aux tiques.

## Abstract (en)

**Introduction:**

New species of *Borrelia*, *Rickettsia*, and *Anaplasma* have recently been detected in ticks in French Guiana. Additionally, a new species of *Anaplasma* was discovered in a human. Specific genovariants of *Anaplasma* and *Ehrlichia* have also been identified in the Amazonian forest of French Guiana, indicating a specific sylvatic cycle.

**Case presentation:**

We present the case of a patient who experienced fever, myalgia, rash, and inoculation eschars following dozens of tick bites while hiking in the Amazon rainforest. Laboratory results showed lymphopenia, neutrophilia, hepatic cytolysis and cholestasis, and a C-reactive protein level of 78 mg/L. Serology tests for HIV, hepatitis A, B, C, and E; cytomegalovirus; syphilis; and Q fever were negative. NS1 antigen, dengue, and leptospirosis PCR tests were also negative. A skin biopsy of an ulcerated papule on the leg revealed a polymorphic dermal inflammatory infiltrate with intramacrophagic Gram-negative bacilli. Blood PCR for *Anaplasma phagocytophilum*, *Borrelia*, *Rickettsia*, and *Babesia* were negative. Serology tests were negative for *Rickettsia conorii* and *R. typhi,* but positive for *A. phagocytophilum*, with weakly positive IgM and positive IgG. Serological results were confirmed by controlled sample. The patient improved with doxycycline treatment.

**Discussion and conclusion:**

Novel species of *Anaplasma* and *Ehrlichia* have been detected in the Amazon rainforest of French Guiana. These novel genovariants differ from other species in the Northern Hemisphere and are difficult to diagnose with molecular tools not designed to detect pathogens differing from known species. While not proven by molecular biology, this case may represent an anaplasmosis or another infection from the Rickettsiales group. This highlights that little is known about the potential for tick-borne diseases in French Guiana. In this context, recommendations for vector control should extend to ticks as well.

## Introduction

Les tiques sont les deuxièmes vecteurs les plus courants de maladies infectieuses humaines dans le monde après les moustiques [16]. Peu de données sont cependant disponibles sur les maladies vectorielles à tiques chez les humains d’une façon générale, mais plus particulièrement en Guyane. Moins fréquemment décrites que la borréliose de Lyme et la borréliose récurrente à tiques, premières maladies vectorielles à tiques de l’hémisphère Nord [15] ou que les rickettsioses, l’anaplasmose et l’ehrlichiose font partie des maladies transmises par les tiques décrites comme « émergentes » [4]. Le genre *Anaplasma*, de l’ordre des Rickettsiales correspond à des bactéries intracellulaires Gram-négatives, transmises par les tiques de la famille des *Ixodidae* aux hôtes vertébrés [17]. Toutes les espèces n’ont pas la même pathogénicité pour les humains. *Anaplasma phagocytophilum*, agent de l’anaplasmose granulocytaire humaine, est l’une des espèces les plus préoccupantes. Elle a été décrite dans les régions tempérées et tropicales du monde entier, infectant généralement les humains dans les zones rurales en contact étroit avec les animaux domestiques dont le bétail. Toutefois, de nouvelles espèces d’*Anaplasma* susceptibles d’infecter les animaux sauvages ont été signalées [17]. En 2022 un cas d’anaplasmose humaine a été décrit chez un orpailleur splénectomisé [7]. L’agent pathogène en cause est une nouvelle espèce, « *Candidatus* Anaplasma sparouinense » , proche phylogénétiquement de « *Candidatus* Anaplasma amazonensis » connu pour infecter les paresseux à deux et trois doigts (mammifères de l’ordre des Pilosa) brésiliens [4]. Des données de surveillance en faune sauvage ont également révélé la présence d’autres espèces putatives et de génovariants avec un potentiel zoonotique inconnu en Amérique du Sud [4]. Une étude récente portant sur 22 espèces de tiques en Guyane, a révélé qu’un tiers de celles-ci était infecté par des espèces de *Rickettsia.* Le typage par séquençage MLST (*MultiLocus Sequencing Typing*) et l’analyse phylogénétique ont permis d’identifier 19 génotypes de *Rickettsia,* mais aucun n’était identique à 100 % des espèces ou des souches de *Rickettsia* déjà connues [2]. Une autre étude a permis de détecter une nouvelle espèce de *Borrelia*, intermédiaire entre les groupes de la borréliose de Lyme et ceux de la borréliose récurrente à tique, dans des tiques présentes sur des passereaux de la région de Cayenne (notamment le manakin auréole *Pipra aureola,* le manakin tijé *Chiroxiphia pareola* ou le grimpar bec-en-coin *Glyphorynchus spirurus*). La pathogénicité de cette bactérie « *Candidatus* Borrelia mahuryensis » transmise par les tiques, est inconnue. Elle pourrait avoir un impact sur un large spectre d’hôtes, y compris les mammifères américains [3]. Une étude récente sur les cas présumés de borréliose de Lyme en Guyane française n’a cependant pour l’instant pas retenu de cas prouvé ayant pu être acquis avec certitude sur le territoire guyanais mais plutôt en France hexagonale [5].

La Guyane est un territoire français ultramarin situé au nord de l’Amérique du Sud, entre le Suriname et le nord du Brésil, avec de nombreuses zones sauvages. Rassemblant 282 espèces de mammifères sauvages volants et non volants, elle a été reconnue comme l’une des régions présentant la plus grande biodiversité au monde [12]. De nombreux jeunes adultes originaires de France hexagonale travaillent ou partent en vacances dans ce territoire pour le tourisme d’aventure. Ces activités conduisent à explorer des zones forestières avec un risque accru de morsures et de maladies transmises par les tiques [8]. En raison de sa grande diversité écologique et de l’augmentation de l’occupation humaine liée aux activités naturalistes, d’orpaillage ou de loisir entraînant des contacts avec la faune sauvage, la Guyane représente un environnement laissant la porte ouverte à la circulation et à la propagation de nouvelles infections [6]. Pendant longtemps, elle a été considérée comme un territoire exempt de maladies transmises par les tiques, mais des observations récentes ont décrit chez les humains une infection par une nouvelle espèce d’*Anaplasma* [7] et le syndrome Alpha-Gal, une allergie à la viande de mammifères, en lien avec des morsures de tiques [9].

Nous décrivons ici un cas probable d’anaplasmose humaine chez un randonneur français parti en trek en forêt guyanaise.

## Observation clinique

Un homme de 33 ans, caucasien et sportif, qui vivait en Guyane depuis 5 ans, s’est présenté au service des urgences de Cayenne, pour fièvre, éruption cutanée et myalgies 10 jours après avoir été mordu par des tiques en forêt. Il avait passé, en pleine saison sèche, 5 jours à Saül, un village isolé au cœur de la forêt amazonienne, exclusivement accessible en avion. Il avait randonné avec un groupe d’amis dans des parties non déboisées et donc exceptionnellement denses de la forêt, en short et en T-shirt. Ses antécédents médicaux comprenaient une dengue six mois auparavant et une infection à Covid-19 un an auparavant. Il a déclaré avoir été mordu par des dizaines de tiques chaque jour au cours de ses randonnées, qu’il retirait chaque soir sans utiliser d’antiseptique ni d’antibiotique. Six jours après son retour à Cayenne, il a présenté un prurit généralisé, puis, au 8ème jour, un érythème et un œdème du mollet gauche, des myalgies et un aspect inflammatoire de certaines morsures. Au 10^e^ jour, devant l’apparition d’une fièvre, il a consulté un médecin généraliste qui lui a prescrit de l’amoxicilline-acide clavulanique. Le lendemain (11^e^ jour), l’extension de l’érythème sur les bras, le décolleté, l’arrière des oreilles et les paumes, l’amène à consulter aux urgences.

**Figure 1:**
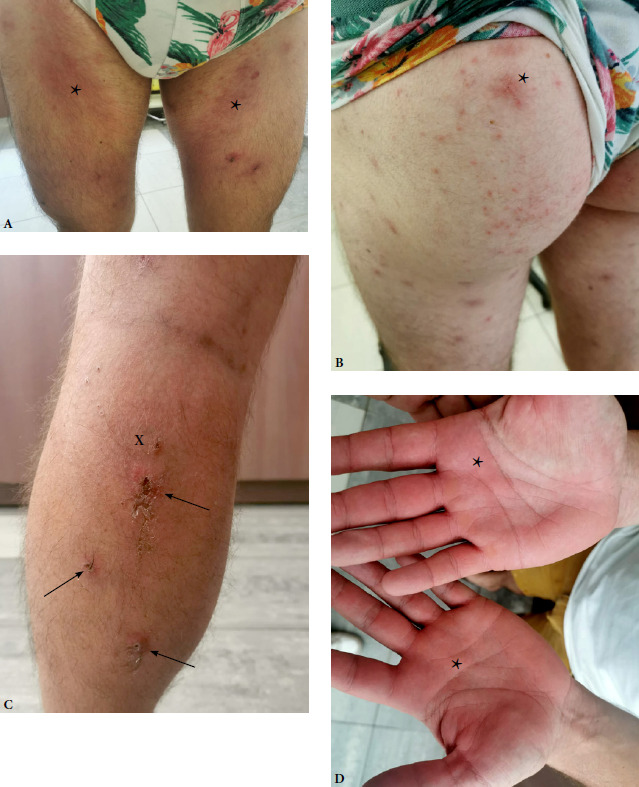
Examen clinique à l’admission. Exanthème maculeux des membres inférieurs (A et B) et palmaire (D) (astérisques), papules ulcéro-crouteuses évoquant des escarres d’inoculation (flèches) et dermohypodermite non nécrosante du mollet gauche (X) (C)

L’examen clinique aux urgences retrouvait un exanthème maculeux (poitrine, bas du dos, face médiale des cuisses), une quinzaine de papules ulcéro-croûteuses des membres inférieurs évoquant des escarres d’inoculation, une dermohy-podermite non nécrosante du mollet gauche, un érythème palmaire et des adénopathies inguinales bilatérales (Fig. 1).

Il n’y avait pas de fièvre mais le patient était sous antibiotique depuis 24h et antipyrétique (paracétamol). Il n’avait pas pris d’anti-inflammatoire non stéroïdien.

Son bilan biologique retrouvait une hémoglobine à 15,2 g/dl, des plaquettes à 324 G/l, des leucocytes à 10 G/l (normes 4-10) avec une polynucléose neutrophile à 7,9 G/l (1,8-7), une lymphopénie modérée à 1,2 G/l (1,5-4) et une protéine C-réactive modérément élevée à 78 mg/l. Une cytolyse hépatique (ASAT 154 UI/l (5N), ALAT 94 UI/l (2N)) et une cholestase (phosphatase alcaline 199 UI/l (1,5N), gamma-glutamyltransférase 340 UI/l (7N)) étaient présentes, sans rhabdomyolyse avec une créatininémie à 83 µmol/l. Les sérologies VIH, hépatites A, C et E (IgG), B (triple négatif), cytomégalovirus (IgG et IgM), syphilis, *Coxiella burnetii* étaient négatives ainsi que l’antigène NS1 et la PCR pour la dengue et la leptospirose.

Le traitement antibiotique par amoxicilline-acide clavulanique 1 g x 3/j a été poursuivi dans le contexte de dermohypodermite et de la doxycycline 100 mg x 2/j a été débutée devant une suspicion de maladie vectorielle à tiques au vu du tableau clinique évocateur, bien que les données épidémiologiques ne soient pas en faveur de telles infections en Guyane.

Le patient s’est rapidement amélioré avec diminution de la cholestase et de la cytolyse (sans correction totale) en quelques jours avec cette bi-antibiothérapie et a pu quitter l’hôpital.

**Figure 2:**
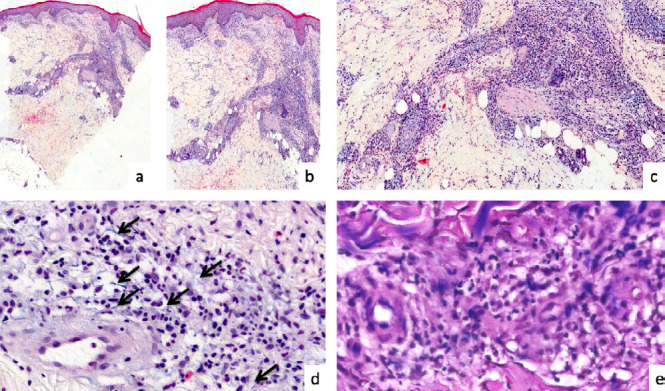
Biopsie cutanée de la cuisse gauche

L’examen anatomopathologique de la biopsie cutanée d’une lésion ulcéro-croûteuse de la cuisse gauche a mis en évidence un infiltrat inflammatoire dermique polymorphe associant des lymphocytes, des polynucléaires éosinophiles et des macrophages de localisation périvasculaire et péri annexielle avec des bacilles Gram-négatif intramacrophagiques en faveur d’une anaplasmose ou d’une rickettsiose (Fig. 2). Des immunomarquages spécifiques pour les bactéries transmises par les tiques n’ont pas pu être réalisés. Après déparaffinage, une analyse en métagénomique nouvelle génération (mNGS) n’a pas permis d’identifier la bactérie.

Un frottis cutané d’une lésion ulcéro-croûteuse retrouvait en culture bactériologique du *Staphylococcus aureus*.

La sérologie *A. phagocytophilum* (réalisée au 12^e^ jour du début des symptômes) était positive avec des IgM faiblement positives et des IgG positives. Les sérologies *R. conorii* et *R. typhi* étaient négatives. Les résultats de la PCR sanguine et sur biopsie cutanée étaient négatifs pour *A. phagocytophilum* . De même, les résultats de la PCR sanguine pour *Borrelia, Rickettsia* et *Babesia* étaient négatifs. Il n’a pas été réalisé de sérologie ni de PCR *Ehrlichia*.

À noter que le patient n’avait pas conservé de tiques qui auraient pu permettre l’identification de celles-ci.

Aucun des autres membres du groupe avec lesquels il voyageait n’avait présenté de symptômes malgré les nombreuses morsures de tiques. La seule différence entre lui et ses compagnons était l’absence d’utilisation régulière d’une désinfection, voire d’une antibiothérapie en cas d’association à un érythème.

L’extension de l’éruption survenue après 24 heures d’amoxicilline-acide clavulanique n’a pas été considérée comme une toxidermie puisque le prurit et les premières lésions étaient déjà présents avant le début du traitement et que les lésions, comme l’anatomopathologie, n’étaient pas en faveur. Lors du suivi ambulatoire, le patient n’a présenté aucune complication. La sérologie de contrôle à 2 mois retrouvait des résultats similaires avec une positivité faible en IgM et forte en IgG (pas de quantification réalisée). Il n’y a pas eu de sérologie de contrôle pour d’autres pathogènes (rickettsiose notamment).

(a) Épiderme légèrement acanthosique (coloration à l’hématoxyline et à l’éosine (HES), x40)

(b) Le derme superficiel est œdémateux avec des globules rouges extravasés (HES, X60)

(c) Le derme est dissocié par un important infiltrat inflammatoire de localisation périvasculaire et péri annexielle riche en macrophages et en éosinophiles (HES, X400).

(d) Les flèches noires indiquent la présence de petites bactéries bleues dans les macrophages (HES, X1000).

(e) Ces bactéries ne sont pas colorées par la coloration de Gram (souche Gram, X1000)

## Discussion

Nous avons présenté ici un cas de myalgies fébriles associées à un exanthème maculeux et à des lésions ulcéro-croûteuses des membres inférieurs après morsures de tiques pour lequel le diagnostic d’anaplasmose a été présumé devant un examen de Gram et une sérologie en faveur, mais présentant des éléments dermatologiques inhabituels.

Si la fièvre et les myalgies sont présentes dans la plupart des maladies transmises par les tiques comme les rickettsioses, l’ehrlichiose ou l’ana-plasmose, l’éruption cutanée est décrite comme rare avec *A. phagocytophilum* mais présente dans 20-88 % des cas avec *Ehrlichia chaffeensis* notamment au niveau des membres et du tronc [14], avec parfois une atteinte palmaire [1]. Les escarres d’inoculation peuvent être décrites en cas d’ehrlichiose [14], mais sont cependant plus souvent rapportées dans les rickettsioses [1].

Au niveau amazonien, dans le cas de l’anaplasmose à « *Ca.* Anaplasma sparouinense »*,* il n’y avait pas de description de signe dermatologique [7]. Le patient a-t-il présenté plusieurs infections transmises par les tiques ou a-t-il présenté un génovariant spécifique « amazonien » avec une présentation clinique à cheval entre les différentes espèces connues actuellement ?

La proximité phylogénétique de « *Ca.* Anaplasma sparouinense *»* avec d’autres *Anaplasma* trouvées chez les tiques, les oiseaux et les mammifères sauvages de la forêt amazonienne montre qu’un groupe génétique d’*Anaplasma* y circule [7]. En 2024, une surveillance approfondie des infections à *Ehrlichia* et *Anaplasma* en forêt amazonienne a trouvé une diversité non documentée auparavant de génovariants d’*Anaplasma* et d’E*hrlichia* circulant chez les tiques et la faune sauvage (mammifères : paresseux, opossums, tatous; oiseaux : passereaux…) de Guyane dans le cadre d’un cycle sylvatique [4]. Beaucoup de ces génovariants n’ont jamais été observés ailleurs, la plupart étant même de nouvelles espèces [4]. L’environnement forestier spécifique de Saül avec une forte densité de zones habituellement préservées de l’activité humaine, a déjà été associé à la découverte d’espèces inhabituelles de *Leishmania* [13] ou du virus Oropouche [10] et pourrait également abriter un type inhabituel d’agents responsables d’anaplasmose.

Les anticorps contre *Anaplasma* sont détectés en moyenne 11,5 jours après l’apparition des symptômes [11], ce qui correspond à notre cas. La sérologie de contrôle, réalisée un peu plus tardivement que dans les recommandations (2 mois au lieu de 2-4 semaines) [1] retrouvait des titres similaires. Il n’a malheureusement pas été possible de réaliser une quantification, car la majoration du titre des IgG aurait été en faveur de notre hypothèse.

L’absence de sérologie initiale pour *Ehrlichia* ni de contrôle pour d’autres infections transmises par les tiques comme la rickettsiose ne permet pas non plus d’avoir des arguments pour un génovariant spécifique de ces pathologies.

Bien qu’aucun test *Gold Standard* n’ait été défini, la PCR sanguine est l’un des tests les plus fiables en raison de sa spécificité élevée [11]. Sa sensibilité est cependant inférieure à celle de la sérologie et diminue après la première semaine de symptômes et 24 à 48 heures après l’instauration des antibiotiques [1]. Dans notre cas, la négativité de la PCR pourrait s’expliquer par le fait que les échantillons sanguins et cutanés ont été prélevés 12 jours après l’apparition des symptômes et après 2 doses de doxycycline. La bactérie pourrait également n’avoir été présente que dans la biopsie cutanée envoyée pour l’anatomopathologie. Cela expliquerait aussi pourquoi elle n’a pas été trouvée sur le frottis cutané qui ne présentait que du *S. aureus* en culture, probablement en lien avec la dermohypodermite secondaire. Il faut rappeler que les valeurs diagnostiques de la PCR ainsi que du frottis cutané sur la biopsie d’escarre sont faibles et ne sont pas reconnues comme un bon outil diagnostic de l’anaplasmose. De plus la culture bactérienne est rarement positive pour les bactéries intracellulaires si des techniques spécifiques ne sont pas utilisées (ce qui n’était pas le cas ici) [1].

Les outils moléculaires ont également pu être limités dans la détection d’un nouveau variant, différent de la forme classique. En effet, ces méthodes ne permettent pas de détecter des espèces différentes de celles déjà connues, ce qui constitue une limitation majeure pour la détection de nouvelles espèces. Si l’on considère que la diversité microbienne de la Guyane est largement inconnue, cela laisse un large éventail d’agents pathogènes potentiels [6].

Il est toutefois bon de préciser que si ce risque existe, il semble assez faible puisque dans notre cas une seule personne du groupe a été infectée.

## Conclusion

Même si elle n’a pas été prouvée par des outils moléculaires, cette présentation épidémiologique, clinique et biologique concordante avec une anaplasmose ou une autre infection par un agent de l’ordre des Rickettsiales, soulève des questions sur le risque de maladies infectieuses transmises par les tiques en Guyane. Avec la description récente d’un cas d’anaplasmose humaine et de syndromes d’Alpha-Gal liés à des morsures de tiques sur ce territoire, une attention particulière doit être portée aux patients présentant des morsures de tiques. Dans ce contexte, les recommandations de protections antivectorielles doivent être étendues aux tiques en Guyane française.

## Considérations éthiques

Le patient a signé un formulaire de consentement pour la publication de son cas ainsi que des photos.

## Financement

Aucun.

## Contribution des auteurs et autrices

MBW : conception de l’étude, collecte des données, figures, analyse des données, recherche documentaire, rédaction et révision; KDA : réalisation des examens biologiques, analyse des données, rédaction et révision; OD : réalisation des examens biologiques, analyse des données, recherche documentaire, rédaction et révision; JD : collecte des données, figures, révision; MD : recherche documentaire, rédaction et révision; LE : conception de l’étude, recherche documentaire, rédaction et révision, et PC : conception de l’étude, analyse des données, recherche documentaire, rédaction et révision.

Tous les auteurs ont lu et approuvé le manuscrit.

## Déclaration de liens d’intérêt

Aucun lien d’intérêt n’a été déclaré.
