# Supplementary material for: Tick-borne disease in a backpacker in French Guiana: one case of presumed anaplasmosis
Source: Med Trop Sante Int. 2026 Mar 13;6(1):mtsi.v6i1.2026.826. [Article in French] doi: 10.48327/mtsi.v6i1.2026.826 (PMC13216906; doi:10.48327/mtsi.v6i1.2026.826)
Supplement: Related texts

# Research article: Tick-borne disease in a backpacker in French Guiana: one case of presumed anaplasmosis

## Introduction

Ticks are the second most common vectors of infectious diseases in humans worldwide, after mosquitoes [16]. However, little data is available on tick-borne diseases in humans, particularly in French Guiana. Defined as "emerging" tick-borne diseases, anaplasmosis and ehrlichiosis are described less frequently than Lyme borreliosis and relapsing tick-borne borreliosis, the leading tick-borne diseases in the Northern Hemisphere [15], or rickettsioses [4]. The genus *Anaplasma*, of the order Rickettsiales, consists of Gram-negative intracellular bacteria that are transmitted by *Ixodidae* ticks to vertebrate hosts [17]. Not all species are equally pathogenic to humans. *Anaplasma phagocytophilum*, the causative agent of human granulocytic anaplasmosis, is one of the most concerning species. It has been found in temperate and tropical regions worldwide, primarily infecting humans in rural areas who are in close contact with domestic animals, including livestock. However, new *Anaplasma* species capable of infecting wild animals have been reported [17]. In 2022, a case of human anaplasmosis was reported in a splenectomized gold miner [7]. The causative pathogen is a new species, “*Candidatus* Anaplasma sparouinense,” which is phylogenetically close to “*Candidatus* Anaplasma amazonensis,” a species that infects Brazilian sloths (mammals of the order Pilosa, both two- and three-toed) [4]. Wildlife surveillance data also revealed the presence of other putative species and genovariants with unknown zoonotic potential in South America [4]. A recent study of 22 tick species in French Guiana revealed that one-third of them were infected with *Rickettsia* species. MultiLocus Sequencing Typing (MLST) and phylogenetic analysis identified 19 *Rickettsia* genotypes, none of which were 100% identical to any known species or strains [2]. Another study identified a new species of *Borrelia* that is intermediate between the Lyme borreliosis and relapsing tick borreliosis groups. This species was found in ticks on passerines in the Cayenne region, including the crimson-hooded manakin *Pipra aureola*, the blue-backed manakin *Chiroxiphia pareola*, and the wedge-billed woodcreeper *Glyphorynchus spirurus*. The pathogenicity of this tick-borne bacterium, “*Candidatus* Borrelia mahuryensis,” is unknown. It could impact a wide range of hosts, including American mammals [3]. However, a recent study of suspected cases of Lyme borreliosis in French Guiana did not identify any confirmed cases acquired in French Guiana, but rather imported from mainland France [5]. French Guiana, a French overseas territory, is located in northern South America between Suriname and northern Brazil, with many unexplored and wild areas. Gathering 282 species of wild flying and non-flying mammals’ species, it has been recognized as one of the regions with the highest biodiversity in the world [12]. Many young adults from mainland France visit this territory for adventure tourism. These activities lead to the exploration of forested areas, which increases the risk of tick bites and tick-borne diseases [8]. Due to its great ecological diversity and the increase in human occupation linked to nature activities, gold mining, and leisure activities involving contact with wildlife, French Guiana is an environment conducive to the circulation and spread of new infections [6]. It was long considered a territory free of tick-borne diseases, but recent observations have reported infection in humans with a new species of *Anaplasma* [7] and alpha-gal syndrome, a mammalian meat allergy linked to tick bites [9]. Here, we present a probable case of human anaplasmosis in a French hiker who went trekking in the French Guiana forest.

## Clinical observation

A 33-year-old Caucasian man who was athletic and had lived in French Guiana for five years presented to the emergency department in Cayenne with a fever, rash, and myalgia. Ten days prior, he had been bitten by ticks in the forest. During the dry season, he spent five days in Saül, an isolated village in the heart of the Amazon rainforest accessible only by plane. While there, he hiked with friends through dense, uncultivated forest areas while wearing shorts and a T-shirt. His medical history included dengue fever six months earlier and a SARS-CoV-2 infection one year earlier. He reported being bitten by dozens of ticks daily during his hikes and removing them each evening without using antiseptic or antibiotics. Six days after returning to Cayenne, he developed generalized pruritus. Then, on the eighth day, he developed erythema and edema of the left calf, myalgia, and an inflammatory aspect of some of the bites. On the 10^th^ day, he developed a fever and consulted a general practitioner, who prescribed amoxicillin-clavulanic acid. The next day, the 11^th^ day, the erythema spread to his arms, neck, the backs of his ears, and palms, leading him to go to the emergency room.

The patient’s clinical examination in the emergency room revealed a macular rash on the chest, lower back, and medial thighs; about fifteen ulcerative, crusted papules on the lower limbs that were suggestive of inoculation eschars; cellulitis of the left calf; palmar erythema; and bilateral inguinal lymphadenopathy (Fig. 1).

The patient did not have a fever, but had been taking antibiotics for 24 hours and antipyretics (paracetamol). He had not taken any nonsteroidal anti-inflammatory drugs.

Laboratory tests showed the following: hemoglobin 15.2 g/dL; platelets 324 G/L; leukocytes 10 G/L (normal range 4-10), with neutrophilic polynucleosis 7.9 G/L (normal range 1.8-7); moderate lymphopenia 1.2 G/L (normal range 1.5-4); and moderately elevated level of C-reactive protein 78 mg/L. Hepatic cytolysis (AST 154 IU/L (5N) and ALT 94 IU/L (2N)) and cholestasis (alkaline phosphatase 199 IU/L (1.5N) and gamma-glutamyltransferase 340 IU/L (7N)) were present without rhabdomyolysis, and creatinine was at 83 µmol/L. Serology tests for HIV, hepatitis A, C, and E (IgG), hepatitis B (triple negative), cytomegalovirus (IgG and IgM), syphilis, and *Coxiella burnetii* were negative, as were NS1 antigen and PCR tests for dengue and leptospirosis.

**Figure 1:** Clinical examination upon admission. Macular rash on the lower limbs (A and B) and palms (D) (asterisks); ulcerative papules suggestive of inoculation eschars (arrows); and cellulitis of the left calf (X) (C)

The patient recieved an antibiotic regimen with amoxicillin-clavulanic acid (1 g three times a day) for cellulitis and doxycycline (100 mg twice a day) was added for suspected tick-borne disease, despite the lack of epidemiological data supporting such infections in French Guiana.

The patient rapidly improved with a reduction in cholestasis and cytolysis (though not complete) within a few days of receiving dual antibiotic therapy, after which he was able to leave the hospital. Pathological examination of a skin biopsy from an ulcerative, crusted lesion on the left thigh revealed a polymorphic dermal inflammatory infiltrate involving lymphocytes, eosinophils, and macrophages that were located perivascularly and periadnexally, as well as an intramacrophagic Gram-negative bacilli, suggestive of anaplasmosis or rickettsiosis (Fig. 2). However, specific immunomarking for tick-borne bacteria could not be performed. After deparaffinization, next-generation metagenomic sequencing (mNGS) failed to identify the bacterium. A skin smear from an ulcerative-crusted lesion revealed *Staphylococcus aureus* in bacteriological culture.

*A. phagocytophilum* serology, performed on the 12^th^ day after symptom onset, was positive with weakly positive IgM and positive IgG. *R. conorii* and *R. typhi* serology were negative. PCR on the blood and skin biopsy were negative for *A. phagocytophilum* . Similarly, blood PCR for *Borrelia*, *Rickettsia*, and *Babesia* were negative. No *Ehrlichia* serology or PCR were performed.

It should be noted that the patient did not save any of the ticks that could have been used for identification.

Despite numerous tick bites, none of the other members of the group with whom he was traveling had any symptoms. The only difference between him and his companions was his failure to undergo regular disinfection or antibiotic therapy in cases of erythema.

The rash that spread after 24 hours of amoxicillin-clavulanic acid treatment was not considered a toxidermia because the pruritus and initial lesions were present before treatment began, and the lesions and pathology were not supportive of this diagnosis. During outpatient follow-up, the patient experienced no complications. The two-month follow-up serology showed similar results, with low IgM and high IgG positivity (no quantification was performed). There was no serology testing for other pathogens, particularly rickettsiosis.

**Figure 2:** Skin biopsy of the left thigh

(a) Slightly acanthotic epidermis (hematoxylin and eosin stain, x40). (b) The superficial dermis is edematous with extravasated red blood cells (H&E stain, x60)

(c) The dermis is infiltrated by a significant inflammatory response located perivascularly and periadnexally. This response is rich in macrophages and eosinophils (H&E stain, x400)

(d) Black arrows indicate small blue bacteria within macrophages (H&E stain, x1000)

(e) These bacteria are not stained by Gram staining (Gram stain, x1000)

## Discussion

We present a case of febrile myalgia accompanied by macular exanthema and ulcerative-crusted lesions on the lower extremities following tick bites. The diagnosis of anaplasmosis was presumed based on Gram staining and serology; however, the case exhibited unusual dermatological features.

Although fever and myalgia are common in tickborne diseases, such as rickettsiosis, ehrlichiosis, and anaplasmosis, skin rashes are rare with *A. phagocytophilum* but present in 20%-88% of cases with *Ehrlichia chaffeensis*, primarily on the limbs and trunk [14], and sometimes involving the palms [1]. Inoculation lesions may be present in cases of ehrlichiosis [14], but they are more commonly reported in rickettsioses [1].

In the Amazon region, in the case of anaplasmosis caused by “*Ca.* Anaplasma sparouinense”, there was no description of dermatological signs [7]. Was the patient infected with multiple tick-borne infections, or did he exhibit a specific “Amazonian” genovariant with a clinical presentation that overlaps with those of the different currently known species?

The phylogenetic proximity of “*Ca.* Anaplasma sparouinense” and other *Anaplasma* species found in ticks, birds, and wild mammals in the Amazon rainforest indicates that a genetic group of *Anaplasma* circulates there [7]. In 2024, extensive surveillance of *Ehrlichia* and *Anaplasma* infections in the Amazon rainforest revealed previously undocumented *Anaplasma* and *Ehrlichia* genovariants circulating in ticks and wildlife, including sloths, opossums, armadillos (mammals), and passerines (birds), as part of a sylvatic cycle in French Guiana [4]. Many of these genovariants have never been observed elsewhere; most of them are new species [4]. The specific forest environment of Saül, which has a high density of areas that are usually preserved from human activity, has been associated with the discovery of unusual species of *Leishmania* [13] and of the Oropouche virus [10]. This environment may also harbor unusual agents that cause anaplasmosis. On average, antibodies against *Anaplasma* are detected 11.5 days after the onset of symptoms [11], which is consistent with our case. The control serology, performed slightly later than recommended (two months instead of two to four weeks) [1], found similar titers. Unfortunately, quantification was not possible, as an increase in the IgG titer would have supported our hypothesis.

The absence of initial serology for *Ehrlichia* and of controls for other tick-borne infections, such as rickettsiosis, prevents us from arguing for a specific genovariant of these pathologies.

Although no Gold Standard test has been defined, blood PCR is one of the most reliable due to its high specificity [11]. However, its sensitivity is lower than serology’s and decreases after the first week of symptoms and 24 to 48 hours after starting antibiotics [1]. In our case, the negative PCR result could be explained by the timing of the blood and skin sample collection: 12 days after symptom onset and after two doses of doxycycline. The bacteria may have been present only in the skin biopsy that was sent for pathological examination. This would also explain why the bacteria was not found in the skin smear, which showed only *S. aureus* in culture, likely due to secondary cellulitis. It should be noted that PCR and skin swabs on eschar biopsies have low diagnostic value and are not recognized as good diagnostic tools for anaplasmosis. Additionally, bacterial culture rarely detects intracellular bacteria unless specific techniques are used, which was not the case here [1].

Molecular tools may also be limited in detecting new variants that differ from the classic form. These methods cannot detect species differing from those already known, which limits their ability to detect new species. Considering the largely unknown microbial diversity of French Guiana, a wide range of potential pathogens remains [6].

However, while this risk exists, it appears to be fairly low since only one person in this group was infected.

## Conclusion

Although not confirmed by molecular testing, this epidemiological, clinical, and biological presentation is consistent with anaplasmosis or another rickettsial infection, raising questions about the risk of tick-borne infectious diseases in French Guiana. Given the recent description of cases of human anaplasmosis and Alpha-Gal syndrome linked to tick bites in this region, special care must be taken with patients who have been bitten by ticks. In this context, recommendations for vector control should extend to ticks in French Guiana.

## Ethical considerations

The patient signed a consent form for the publication of his case and photos.

## Funding

None.

## Authors’ contributions

MBW: study design, data collection, figures, data analysis, literature review, writing and revision; KDA: biological testing, data analysis, writing and revision; OD: biological testing, data analysis, literature review, writing and revision; JD: data collection, figures, revision; MD: literature review, writing and revision; LE: study design, literature review, writing and revision; PC: study design, data analysis, literature review, writing and revision.

All authors have read and approved the manuscript.

## Conflicts of interest

The authors declare no conflicts of interest related to this work.
